# High-Risk Human Papillomaviral Oncogenes E6 and E7 Target Key Cellular Pathways to Achieve Oncogenesis

**DOI:** 10.3390/ijms19061706

**Published:** 2018-06-08

**Authors:** Nicole S. L. Yeo-Teh, Yoshiaki Ito, Sudhakar Jha

**Affiliations:** 1 Cancer Science Institute of Singapore, National University of Singapore, Singapore 117599, Singapore; nicole.yeo@u.nus.edu (N.S.L.Y.-T.); yoshi_ito@nus.edu.sg (Y.I.); 2 NUS Graduate School for Integrative Sciences and Engineering, National University of Singapore, Singapore 117456, Singapore; 3 Department of Biochemistry, Yong Loo Lin School of Medicine, National University of Singapore, Singapore 117596, Singapore

**Keywords:** HPV, human papillomavirus, cervical cancer, viral oncogenes, E6, E7, viral-induced cancers

## Abstract

Infection with high-risk human papillomavirus (HPV) has been linked to several human cancers, the most prominent of which is cervical cancer. The integration of the viral genome into the host genome is one of the manners in which the viral oncogenes E6 and E7 achieve persistent expression. The most well-studied cellular targets of the viral oncogenes E6 and E7 are p53 and pRb, respectively. However, recent research has demonstrated the ability of these two viral factors to target many more cellular factors, including proteins which regulate epigenetic marks and splicing changes in the cell. These have the ability to exert a global change, which eventually culminates to uncontrolled proliferation and carcinogenesis.

## 1. Introduction

In 1966, Dr. Peyton Rous was awarded the Nobel Prize in Medicine and Physiology for his discovery of the Rous sarcoma virus, which has the ability to cause sarcoma in chickens, although his discovery was made five decades earlier in 1911 [1]. This was mainly due to the great amount of skepticism that his work was met with, and the rejection of using an avian model to study infectious cancers in human. Since then, there has been a growing interest in identifying the role of viruses in cancer. Currently, it is estimated that of all cancers worldwide, 20–25% have a viral etiology [2]. There are seven known viruses which can cause cancer in humans—DNA tumor viruses comprising of human papillomavirus (HPV), Epstein–Barr virus (EBV), Kaposi’s sarcoma-associated herpesvirus (KSHV), Merkel cell polyomavirus (MCV), hepatitis B virus (HBV), and RNA viruses which include hepatitis C virus (HCV) and human T-lymphotropic virus-1 (HTLV-1).

HPVs can also be classified as cutaneous or mucosal type, depending on its target tissue. Cutaneous HPV types infect the basal epithelial cells of the hands and feet (and typically cause plantar warts, common warts, and flat warts (reviewed in [3]), while mucosal types infect the inner lining of tissues, such as the respiratory tract [4], oropharyngeal region [5], or anogenital epithelium. The mucosal types can be further divided to high-risk or low-risk HPV, and it is the high-risk mucosal HPV types which have been found to have close links with cervical cancer.

HPV is a double-stranded circular DNA virus, between 6.8 kb and 8 kb in length, and is the most common sexually-transmitted infection. There are now over 200 subtypes of the virus, which can be categorized as either low-risk or high-risk, depending on their activity on the host cell. The difference between high and low-risk HPV lies mainly in their cellular targets, wherein the targets of high-risk HPV have the ability to cause the formation of tumors while the cellular targets of low-risk HPV are not able to. This review, however, will focus on the cellular pathways targeted by high-risk HPV.

In 1983, the first high-risk HPV type (HPV16) was discovered by a German virologist and a Nobel Prize awardee (in 2008), Professor Harald zur Hausen, when he attempted to find similarities in DNA sequences between patients with cervical cancer [6]. In 61.1% of cervical cancer samples, the extracted DNA hybridized to a HPV16 probe under highly stringent conditions, while lower hybridization rates were obtained from samples from other diseased tissue, alluding to a possible causative function of HPV in cervical cancer. Since then, 13 high-risk types (types 16, 18, 31, 33, 35, 39, 45, 51, 52, 56, 58, 59, and 68) have been discovered, with HPV16 and 18 being the most common in patients with invasive cervical carcinomas.

### 1.1. HPV in Cancers

Although the presence of the HPV genome has been found in cancers of multiple tissue origins (see references above), the strongest link of HPV has been in cancer of the cervix. Cervical cancer is the fourth most common cancer in females worldwide, behind breast, colon, and lung cancer, with a similar standing in mortality rate [7]. There is variation in the incidence and mortality rate depending on geographical location, and this is primarily due to the difference in education of prevention and availability to screening. An estimated 530,000 cases of invasive cervical cancer are diagnosed globally every year [8]. Of these, 99.7% of samples have tested positive for HPV [9,10]. The absence of HPV in the remaining 0.3% of cervical carcinomas has been attributed to the following factors: inadequate detection methods, presence of unidentified types of HPV, and disruption of the HPV genome during integration events [11].

HPV has also been implicated heavily in head and neck squamous cell carcinoma (HNSCC), with the virus found in up to 25% of patients [12,13]. Since the etiological link between HPV and HNSCC was made in 1983, there has been a significant increase in HPV-positive HNSCC samples detected. Similar to cervical cancer, the most common type of HPV known to infect HNSCC patients is HPV16, accounting for over 80% of HPV-positive HNSCC patients. Other cancers which have been linked to HPV include breast [14,15,16], anal, vaginal, penile, and vulvar [17]. However, in these cancers, the incidence of HPV is variable, with relatively lower detectable viral load. Specific techniques have been used to detect the low level of HPV in these samples. These techniques include in situ PCR [18,19], histology [14], bead-based Luminex technology [20], hybrid capture 2 (HC2) assay [21], and HPV capture paired with massive paralleled sequencing [22]. The low levels of HPV detected in these patients does not discount HPV from having a causative role. Instead, it has been hypothesized that there are other factors which could drive the cancer in HPV-negative patients. This review, however, will focus on HPV in cervical cancer.

### 1.2. HPV Genome

There are three distinct regions in the HPV genome: the early gene-coding region (E), late gene-coding region (L), and the long control region (LCR). The early genes (E1, E2, E4–E7) encode for viral replication and regulatory proteins, three of which, E5, E6, and E7, are oncogenic. Depending on their specific function, the early genes are expressed at different stages of the viral life cycle [23,24]. The late genes encode for two structural proteins involved in the formation of the viral capsid, while the LCR contains regulatory elements involved in the control of viral DNA replication and transcription [23,24].

Upon HPV infection of cells in the basal layer, E1 and E2 facilitate viral genome replication at a low-copy number rate. However, it is not until the basal cells differentiate into the suprabasal layer of the epithelia prior to sloughing off that the virus switches to high-copy replication rate. Although the exact mechanism of virion release is not known, it occurs at around the same time as epithelia desquamation. These virions are then capable of infecting other neighboring cells. Despite the fact that integration of the HPV genome can occur in pre-malignant lesions, the proportion of cells which undergo HPV integration increases as the cells progress in malignancy [25]. One of the first proposed mechanisms of integration demonstrated that the breakpoint of circular HPV episome was at the E2 gene [26]. This was of great interest as E2 transcriptionally represses E6 and E7, and therefore, the disruption of E2 upon integration leads to the de-repression of E6 and E7. In cells where the HPV genome was integrated, most viral genes are found to be silenced, with the exception of E6 and E7.

Since the integration of the viral genome into the host is a “dead-end” of the viral life cycle, it seems counterintuitive that the virus would integrate, and in doing so, limiting its infectious potential. However, scientists have rationalized this, and since a virus lacks machinery to replicate its own genome, it needs to hijack its host’s replication machinery [27]. The driving oncogenes, E6 and E7, hijack the cellular ubiquitin–proteasome system (UPS) to degrade retinoblastoma protein (pRb), forcing the host cell into S phase, where replication machinery and resources, such as nucleotide pool, are abundant. This allows viral replication to occur [28]. Since unscheduled cell cycle progression would trigger apoptosis in the cell, HPV E6 concurrently degrades p53, which would result in the evasion of apoptosis [29]. Through the constitutive expression of E6 and E7, coupled with aneuploidy that is brought on by uncontrolled cell division, genomic instability ensues. This provides the cells a growth advantage and promotes oncogenesis [30]. Therefore, the ultimate aim of the virus is not to cause carcinogenesis in the host organism. Instead, the formation of tumors is an unwanted, accidental side effect of viral infection.

Despite the growth advantage that follows an integration event, not all HPV-positive cells undergo integration. A study analyzing HPV-positive samples obtained from The Cancer Genome Atlas revealed integration events in all HPV18-positive samples, but only occurred in 76% of HPV16-positive samples [31]. However, it is interesting to note that in samples where the HPV genome is not integrated, there are often epigenetic or genetic changes found on the regulatory regions of E6 and E7 that result in the dysregulation of these two oncogenes [32,33,34]. This indicates that although cancer progression and the integration of HPV genome are separate events, the dysregulation of E6 and E7 is crucial for tumorigenesis.

### 1.3. HPV Oncogenes

Although all the genes encoded by the HPV genome are crucial during different stages of replication and the viral life cycle, the two most important genes in high-risk HPV are E6 and E7, also known as the key viral oncogenes. They were termed viral oncogenes due to their targets in the cell and the implications of this dysregulation. As discussed earlier, although the main target of E6 and E7 are p53 [29,35] and pRb respectively, much research has shown that there are many other targets of E6 and E7 which allow high-risk HPV types to be tumorigenic. Studies have shown that HPV16 E6 alone is able to immortalize human mammary epithelial cell, allowing it to overcome mortality stage mechanism M1, that is, evade senescence and exhibit an extended lifespan [36]. However, it was found that the overexpression of only E6 and E7 were insufficient for complete cellular transformation in primary human keratinocytes [37].

E5 is the least-studied of all three oncogenes, and was recently shown to contribute to cell cycle progression and unscheduled host DNA synthesis in differentiating keratinocytes [38]. This was shown to be dependent on the EGFR pathway in a ligand-dependent manner, where it functioned to enhance the activation of the EGFR pathway [39,40]. Overexpression of E5 in transgenic mice resulted in hyperplasia of the epithelia, which was rescued by the expression of a dominant negative form of EGFR [41]. High-risk E5 was also shown to enhance immortalization potential of E6 and E7 in keratinocytes [42]. However, it was found that E5 expression is often lost upon HPV integration into the host genome, and therefore, it is postulated that E5 has an important role in the initial stages of cervical carcinogenesis, but less so in its persistence [38].

In following sections, we will discuss the mechanism by which HPV can regulate cellular pathways, primarily through E6 and E7.

## 2. Mechanism of E6 and E7 Regulating Cellular Pathways

### 2.1. Protein Targets Dependent on E6AP

**Figure 1:**
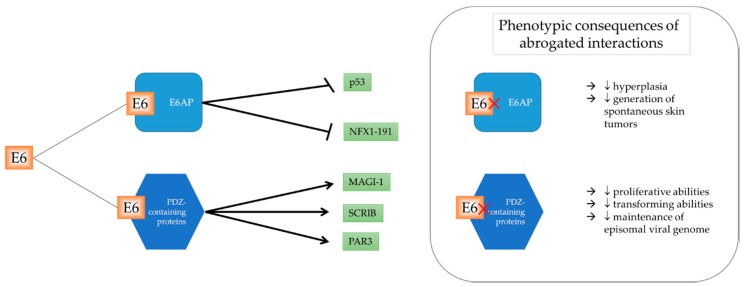
E6 regulates many cellular targets through E6AP and E6′s PDZ-binding motif. Some of these targets are depicted above. Upon the abrogation of the sites on E6 which are involved in the interaction of E6AP and PDZ-containing proteins respectively, there are drastic phenotypic consequences.

HPV regulates cellular pathways through various mechanisms to exert an effect in the infected cell. A summary of E6 regulating selected cellular factors and the consequences of this disruption is illustrated in Figure 1. E6-associated protein (E6AP) is encoded by the *UBE3A* gene and is the founding member of the HECT (homologous to E6AP carboxyl-terminus) E3 family of ubiquitin ligases. E6, in cooperation with E6AP, is one of the most well-studied mechanisms in which HPV degrades its targets. E6AP is a cellular protein, the dysregulation of which has been implicated in Angelman syndrome (AS), a severe neurodevelopmental disorder [43]. However, in the context of HPV-infected cells, E6AP is hijacked by E6 to ubiquitylate and target cellular proteins for degradation by the UPS.

The most prominent target of E6AP is the crucial cellular regulator, p53, more candidly known as the *guardian of the genome* [29,44,45]. E6 binds to the LxxLL consensus sequence in the conserved domain of E6AP, and as a heterodimer, is able to degrade p53 [46]. The structure of the interaction between E6, E6AP, and p53 was solved in 2016 [46]. The interaction of E6 and E6AP was shown to be crucial in vivo, with a transgenic mouse harboring an E6 mutant incapable of binding to E6AP demonstrating reduced levels of skin hyperplasia, and lower generation of spontaneous skin tumors when compared to wild-type E6 [47]. Similar studies demonstrating the importance of E6AP we also carried out, with the transient knockdown of E6AP producing an almost identical phenotype to that of E6 depletion [48]. However, there are an increasing number of studies exploring E6AP-independent manners in which HPV can regulate cellular mechanisms.

### 2.2. PDZ-Domain Family of E6 Interactors

One of the key differences between high-risk and low-risk HPV E6 oncoprotein is the existence of a PDZ (PSD-95/DLG/ZO-1) binding motif (PBM) in high-risk but not low-risk HPV types. The PBM enables the interaction and subsequent degradation of proteins containing a PDZ domain, which is frequently (but not always) located on the C-terminus of proteins [49]. The importance of a PBM on high-risk E6 was demonstrated through a compromised proliferative phenotype in human foreskin keratinocytes (HFKs) containing HPV18 E6ΔPBM when compared to wild-type E6 [50]. Further, cells expressing wild-type E6 demonstrated a selective growth advantage, where extensive passaging resulted in the loss of cells containing E6ΔPBM genome [50]. Interestingly, it was found that both wild-type E6 and E6ΔPBM were able to target and degrade p53, evidenced by a similar response to radiation, due to the lack of p53 and subsequent p21 induction [51].

To date, high-risk E6 is known to interact with at least 14 cellular PDZ-containing proteins, which results in the eventual degradation of the cellular substrate. Interestingly, MAGI-1 is the only cellular target which is degraded by both HPV16 and 18, while the degradation of other cellular substrates is dependent on HPV type [52,53]. MAGI-1 is a member of the MAGI family, which has canonical roles in the regulation of tight junction assembly [54]. Thus, the degradation of MAGI-1 results in the loss of tight junction formation.

Additionally, the interaction of E6 with its PDZ-containing substrates is key in different stages of its viral life cycle, and also in the final stage of oncogenesis. The presence of an intact and functional PBM is seen to be crucial early in the viral life cycle, essential for maintenance of viral copy number and proliferation [55]. Further, E6 PBM is required for the maintenance of episomal viral genome, indicative of its role in viral DNA replication [50]. During oncogenesis, E6’s ability to bind to PDZ domain-containing targets was shown to be important for the host cell’s transformation abilities, both in vitro [56,57] and in vivo [51].

One of the phenotypic changes of immortalized keratinocytes with HPV18 E6 is the marked changes in cell morphology, organization of microfilament network, and the formation of intercellular adhesion junctions [56]. This was shown to be highly, but not completely dependent on a functional PDZ domain. Part of this phenotype can be attributed to the downregulation of Dlg, a cellular target of E6 PDZ-binding motif.

Another study compared the tumorigenic ability of a E6Δ151 mutant (incapable of binding to PDZ domains) to wild-type E6, and showed that the mutant lost almost all tumorigenic ability [58]. This was surprising, since the mutant defective for p53 degradation showed a phenotype close to wild-type E6. The involvement of E6 PDZ substrates was demonstrated when cells containing E6Δ151 mutant were depleted of SCRIB, MAGI-1, and PAR3. While the single knockdown of SCRIB, MAGI-1, or PAR3 only partially but significantly restored tumorigenic ability, co-depletion of SCRIB and MAGI-1 completely restored tumorigenic ability [58].

The members of the 14-3-3 protein family function as adapter proteins, and interact with many cellular proteins with roles in cell cycle, apoptosis, metabolic control, cytoskeletal maintenance, transcription, and tumor suppression [59]. 14-3-3 zeta (an isoform of 14-3-3) is known to interact with E6 in a phospho-specific manner, only interacting with high-risk E6 when its PBM is phosphorylated. This interaction is of particular interest as it is important in maintaining E6 stability in HeLa cells [60]. However, it is still not known whether the phosphorylation of E6’s PBM is required for its interaction with 14-3-3, or whether it is 14-3-3 that modulates the phosphorylation on the PBM of E6.

Although many groups have studied the cellular ubiquitin ligases which are involved in the degradation of cellular PDZ domain-containing proteins, there is little consensus on the mechanism of degradation. Multiple groups have published results showing conflicting data on whether E6AP is involved in the process of degradation, as with Scrib and Bak (targeted by E6AP [61]), while Dlg, MAGI-1, and MUPP1 are not targeted by E6AP [62,63]).

From the evidence presented above, the interaction of E6 with its PDZ domain-containing substrates are very important. Equally as important, perhaps, is the regulation of the PBM on E6. A threonine residue at position 156 within the PBM of E6 is susceptible to phosphorylation by a range of different kinases, which abrogates its ability to fit into the PDZ domain of interactors. Interestingly, it was shown that the PBM of different high-risk HPV types are phosphorylated by different kinases—18E6 by protein kinase A (PKA) and 16E6 by PKA or AKT [60]. However, the phosphorylation of PBM does not always result in the inhibition of interactions.

### 2.3. Other Protein Targets

Since much research has shown E6’s preference to binding to ubiquitin ligases, Poirson et al. screened for novel ubiquitin ligases bound by E6 and E7 [64]. They utilized the *Gaussia princeps* luciferase protein complementation assay (GPCA) to co-express E6 and E7 independently, with a library containing 50% (575 unique protein entries) of the human ubiquitination system. In addition to discovering novel binding partners of E6 and E7, they were also able to study the domains required for interaction. Aside from E6AP, E6 interacting with other ligase components did not require the LxxLL motif, but instead, its PBM on its C-terminus. E7 was also shown to be capable of binding to proteins containing the BTB (BR-C, ttk and bab) domain, RING domain, and deubiquitinating enzymes (DUBs). Furthermore, since the group expressed the HPV oncoproteins from six different HPV types (three high-risk types—HPV16, HPV18, HPV33, one low-risk type—HPV6, and two cutaneous HPV types—HPV8, HPV38), they were able to identify preferential binding of cellular proteins to HPV proteins of different types. In short, they concluded that UPS factors were more differentially bound to E6 than E7, and this was observed across multiple types.

### 2.4. Epigenetic Targets

#### E6/E7 Regulating Methylation Status

Several studies have also shown a close relationship between HPV infection of cells and promoter hypermethylation of cellular genes, repressing their transcription [65,66]. The phenomenon of loci-specific methylation has long been observed in cells infected with HPV, with 87% of CpG sites of methylation variable positions (MVP) showing increased methylation in virus-positive compared to virus-negative cells. Through the comparison of a heterogeneous population HNSCC, it was observed that HPV could modulate the epigenome [66].

In support of the increase in global methylation upon infection with E6/E7, human keratinocytes transduced with E6/E7 were subjected to methylation-specific digital karyotyping (MSDK) and there were 34 genes showing increased methylation [67]. From publicly available gene expression data, the authors showed that these 34 genes had a concomitant decrease in expression. Subsequent validations were successfully performed on a subset of these 34 genes. Although not explored in the paper, it would have been interesting to study the genes that underwent hypomethylation. Part of the mechanism was elucidated when it was shown that transient depletion of E6 resulted in a decrease in DNMT1 protein levels in HPV-positive cell lines in a p53-dependent manner [68].

E-cadherin, in particular, is a functionally significant methylation target of HPV due to its importance in the detection of virus in the epithelia. E-cadherin is expressed on the membrane of keratinocytes, and its presence, together with other stimulatory signals, orchestrates the movement of Langerhans cells through the epithelium, which serves as an endogenous surveillance mechanism [69]. Langerhans cells detect, process, and present viral foreign antigens to other immunocytes, which encourages the stimulation of the host immune system to rid the virus from the organism [69]. In HPV-infected cells, the E-cadherin expression was lower [70], resulting in decreased migration of Langerhans cells [71], and therefore, lower virus clearance rate from the epithelia.

Interestingly, both HPV16 E6 and E7 were found to downregulate *E-cadherin* through DNMT1 activity. The overexpression of HPV16 E6, resulted in decreased surface and total protein level of E-cadherin, as well as an increase in DNMT activity [72]. Although *E-cadherin* levels were rescued after the treatment with DNMT inhibitor 5-aza-2′-deoxycytidine, there was no change in methylation status of E-cadherin promoter in the presence and absence of HPV16 E6. Instead, the authors postulated that HPV16 E6 regulating *E-cadherin* levels was due to the increased presence of repressive elements on E-cadherin promoter, instead. The regulation of DNMT1 by E7 was shown to be via a direct interaction, utilizing the CR3 (conserved region 3) zinc-finger domain of E7, one of the three regions conserved with adenovirus E1A [73]. Although the expression of *E-cadherin* was rescued in normal immortalized human keratinocytes (NIKs) positive for HPV upon the treatment with 5-aza-2′-deoxycytidine, there was no significant change in the methylation status of *E-cadherin* promoter [74]. This led the authors to believe that E7 stabilizes DNMT1 via binding to it, and this regulates another intermediary factor (still unknown), which then leads to the silencing of *E-cadherin.*

The direct effect of E7 and DNMT1, however, was seen in on the promoter of *CCNA1*. This interaction was validated in vitro through ChIP experiments where both DNMT1 and HPV16 E7 were found to be localized on the *CCNA1* promoter, resulting in a repression of *CCNA1* [75]. Following this discovery, it was suggested to utilize the methylation status of *CCNA1* as a potential biomarker to distinguish between normal cervix, low-grade, and high-grade squamous intraepithelial lesions [76,77]. *CXCL14* was also identified as a methylation target of E7, with the HPV oncogene resulting in promoter hypermethylation, and thus, silenced expression [78]. Since CXCL14 has a role in angiogenesis suppression, E7 functions to de-repress angiogenesis to support tumor growth.

Despite numerous studies which have demonstrated HPV’s ability to increase methylation, there have also been a handful of studies which have demonstrated the decrease in methylation at specific loci. For an example, Yin et al. demonstrated that HPV16 E7 led to an increase in the expression of *STK31* [79], a gene which has been implicated in the maintenance of the undifferentiated state of colon cancer cells [80]. Through the use of bisulfite genome sequencing (BGS) PCR and TA cloning, the promoter of *STK31* was shown to be hypomethylated in HPV-infected cells compared to HPV-negative cells. However, the exact mechanism of E7 causing a hypomethylation of the promoter remains to be elucidated. A comparison of tissues from different grades of cervical lesions showed a global hypomethylation upon cervical cancer progression [81]. Through staining with an antibody against methylated CpG, there were no observable differences between normal tissue, benign lesions, low-grade lesions, and high-grade lesions. However, there was a significant decrease in methylation when tissues from invasive SCC were compared to pre-neoplastic lesions.

### 2.5. E6/E7 Targeting Epiegnetic Modifiers

**Figure 2:**
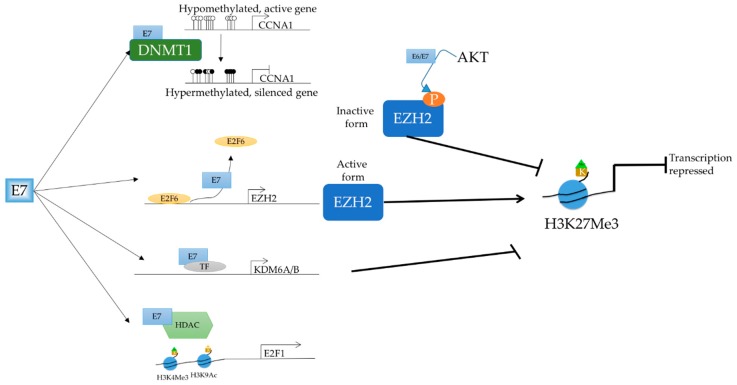
The HPV oncogene E7 is able to regulate multiple epigenetic factors such as DNMT1, HDAC1, and EZH2. E7 associates with DNMT1 on the promoter region of *CCNA1*, resulting in its hypermethylation, and therefore repression. E7 is also able to regulate the repressive H3K27Me3 mark through several of its upstream factors. By binding to the repressive E2F6, E7 is able to de-repress the transcription of the methyltransferase *EZH2*. Conversely, E7 was shown to transcriptionally upregulate *KDM6A/B*, demethylases of H3K27Me3. Further, E6 and E7 has also been shown to recruit AKT to phosphorylate and therefore inactivate EZH2. Lastly, E7 is able to cooperate with HDAC, resulting in the tri-methylation of H3K4 and the acetylation of H3K9 on the promoter of *E2F1* to upregulate the transcription of *E2F1*.

In addition to regulating the methylation status of cellular targets, E6/E7 is also able to interact with other epigenetic modulators which dictate the post-translational modifications on histones, which is illustrated in Figure 2.

There exists an intricate relationship between HPV and polycomb repressive complex (PRC). The two most prominent PRCs are PRC1 and PRC2, which have functions in epigenetic silencing. It has been shown that the HPV oncoproteins interact with different regulators and components of the PRC2 complex, resulting in an altered epigenetic program and the concomitant dysregulation of downstream expression. The EZH2 methyltransferase is the enzymatic component of PRC2, responsible for the tri-methylation of H3K27, a repressive promoter mark. HPV16 E7 ablates the repressive activity of E2F6, a non-canonical member of the E2F family, and transcriptional repressor of EZH2 [82], resulting in the increase in EZH2 expression [83]. However, the increase in EZH2 expression was not shown to result in an increase in global H3K27Me3; instead, it led to a marginal decrease in global H3K27Me3 [84,85]. To date, there are two explanations for this. Firstly, it is known that KDM6A and KDM6B, two demethylases of H3K27Me3, are transcriptionally upregulated by HPV E7 [85]. Secondly, the activity of EZH2 is negatively regulated by phosphorylation by AKT on serine residue 21 [86]. Since both HPV16 E6 and E7 have been shown to activate AKT, the transcription of EZH2 might be upregulated but the activity suppressed [87,88].

Histone acetylation is one of the key post-translational modifications, as it not only signals for its downstream genes to be activated or repressed, but also determines the 3D chromatin structure, defining its euchromatic or heterochromatic state. This is made possible as the acetylation group is able to neutralize the positive charge of histones, therefore loosening the conformation of chromatin, enabling transcription machinery to bind. Although acetylated chromatin is generally a mark of actively transcribed genes, there are instances where an acetylated histone recruits repressive machinery. An example of this is the acetylated histone being read by Brd4, and thus resulting in a repression of downstream gene expression [89,90,91].

HPV has been shown to target both groups of enzymes which are responsible for the deposition and the removal of the acetylated marks—histone acetyltransferases (HATs) and histone deacetylases (HDAC). CBP/p300 is a HAT which acetylates all four canonical histones as well as non-histone targets, and is possibly the most well-studied HAT involved in HPV-mediated changes in epigenetic landscape. The interaction of CBP/p300 and E6 was first shown in 1999 through experiments with purified CBP/p300 and E6 on an affinity column [92]. By sequentially abolishing individual domains of the respective proteins, the E6-binding domain on CBP and p300 was found to be a 19 amino-acid sequence which interacted with the C-terminal zinc finger on E6 [92]. The authors termed it a transcriptional adapter motif (TRAM), which was the same motif targeted by the adenovirus E1A protein [93]. Since CBP/p300 has many cellular targets in the cell, it is most advantageous of E6 to target CBP/p300. One key function of CBP/p300 is as a transcriptional coactivator in p53-dependent transcription, and through its inhibition of CBP/p300, HPV16 E6 is able to attenuate p53 transcription in a E6AP-independent manner [92].

Another target of E6 is the *bona fide* tumor suppressor, TIP60 [89]. This HAT was found to be destabilized by both high-risk and low-risk HPV through the E3 ubiquitin ligase, EDD1, in a proteasome-dependent manner [94]. E6 targeting TIP60 is one element of a feedback loop, since TIP60 is responsible for acetylating histone H4 on the regulatory promoter of E6, thereby recruiting Brd4 to suppress E6 transcription [89].

E6 of high-risk HPV was also shown to regulate the activity of histone methyltransferases (HMTs) co-activator associated arginine methyltransferase 1 (CARM1), protein arginine methyltransferase 1 (PRMT1), and SET domain containing lysine methyltransferase 7 (SET7), thereby affecting p53 levels independent of E6AP [95]. Canonically, CARM1 and PRMT1 are recruited to p53-responsive promoters to activate downstream transcription. However, in the presence of E6, the recruitment and subsequent activity of the two HMTs was abrogated independently of E6 degrading p53. SET7 is a lysine-specific histone methyltransferase that has a canonical function of stabilizing p53 by methylating it at K372, therefore preventing its ubiquitylation and subsequent degradation. However, in the presence of E6, SET7’s enzymatic activity is dampened, subjecting p53 to degradation independent of E6AP [95]. Interestingly, the activity of E6 in regulating TIP60 and the three abovementioned HMTs was not restricted to high-risk HPV, as a similar observation was made with low-risk HPV.

The E7 oncoprotein of HPV has been shown to regulate multiple cellular HDACs through multiple pathways in manners independent and dependent on pRb. Through its interaction with Mi2β, the ATPase subunit of the NuRD complex, E7, is able to bind to HDAC1 through the oncoprotein’s zinc finger domain and attenuate its activity [96,97]. However, when this interaction was abrogated, E7 was no longer able to extend the lifespan of transfected keratinocytes [97].

The family of E2F transcription factors regulate transcription in both positive and negative manners, and have important functions in the cell [98]. E2F2, an activating member of the E2F family, has roles in positively regulating viral genome replication during the process of keratinocyte differentiation, and was also shown to be regulated by HPV 31 E7 [99]. By inhibiting the binding of class I HDACs to the regulatory region of *E2F2*, E7 is able to increase *E2F2* transcription, and therefore promote virus replication. Indeed, the transient depletion of *E2F2* via siRNA resulted in decreased virus replication, although there was no significant change in cell proliferation. The authors concluded that E7 regulating *E2F2* was independent of cell cycle changes mediated by pRb, since the expression of other cell cycle-dependent genes in E2F family did not change.

In a similar manner, E7 was also shown to upregulate the acetylation of H3K9 in a manner dependent on pRb [100]. This was coupled with the tri-methylation of H3K4, a mark of an active promoter, which usually results in the displacement of repressive HDACs from the promoter region [101]. Through mutational analyses, the association with pRb and HDAC was shown to be essential for the post-translational modification of histones, which appeared to be specific to E2F-associated genes.

### 2.6. RNA Targets of HPV

There are many RNA species in the cell, and among other biologically significant molecules, RNA has been proven to have a range of different but important functions in the cell, such as coding for proteins and in modulating coding genes, as in the case of lncRNAs. As such, it comes as no surprise that HPV, too, targets RNA in cells. Two HPV16 proteins, E2 and E6, were shown to have RNA-binding capabilities, both of which negatively affect the splicing of genes with suboptimal intronic splice sites [102]. Interestingly, on the HPV16 E6 protein, the nuclear localization signal 3 (NLS3) at the C-terminus is involved in RNA binding, while a separate N-terminus suppresses RNA splicing. The HPV16 E2 protein, on the other hand, interacts with RNA through its C-terminal DNA binding domain, but utilizes its N-terminal half and hinge region for splicing suppression. Using recombinant HPV proteins and various RNA substrates, the authors demonstrated that HPV16 E6E7 pre-mRNA, BPV-1 late pre-mRNA, and *doublesex* gene from *Drosophila* (all of which contain suboptimal splice sites) had less efficient splicing in the presence of HPV16 E6, while β-globin was spliced efficiently, since it contained optimal splice sites. The mechanism of splicing regulation was partially elucidated when the same authors showed that E2 and E6 bound and interacted with a small subset of splicing regulatory (SR) proteins, although no functional studies were carried out to ascertain this.

Several years later, another group demonstrated the splicing capabilities of HPV16 E6 overexpression in HPV-negative cells [103]. The changes to the transcriptome and splicing profile were compared upon the transient overexpression of HPV16 E6, and validated in clinical tissue samples. It was noted that there was a modest 56 annotated and 22 novel genes which were differentially expressed. Of these, there were 153 skipped exons, 23 alternative 5′ splice sites, 32 alternative 3′ splice sites, and 20 retained introns, although no functional conclusions were drawn.

MicroRNAs (miRNAs) are an abundant class of short RNAs which have the ability to recruit machinery that degrade mature RNA strands, therefore affecting their stability. Depending on its sequence and seed site recognition, different miRNAs have different cellular targets in the cell, with a myriad of consequences. Many studies have been published on miRNAs regulated by HPV, but only a handful of groups have studied miRNAs encoded by HPV itself. To our knowledge, Qian et al. was the first group to experimentally detect miRNAs encoded by HPV [104]. They utilized SOLiD 4 sequencing technology to identify short RNAs in HPV16, HPV38, and HPV68 infected cells, and mirSeqNovel to identify miRNAs encoded specifically by HPV after mapping it to the viral genome. Nine putative miRNAs were detected, of which four were successfully validated via TaqMan assays in cell lines and cervical tissue samples. The authors focused on two miRNAs (HPV16-miR-H1 and HPV16-miR-H2) encoded by HPV16, since it is the most abundant in patients. These miRNAs mapped back to the E1 gene and LCR, respectively. The cellular targets of the two abovementioned miRNAs were predicted based on seed sequence, and were implicated in important host cell processes, such as cell cycle, immune functions, neoplastic development, focal adhesion, cell migration, epithelium development, and cancer. One very interesting cellular gene targeted by both HPV16-miR-H1 and HPV16-miR-H2 is *CYP26B1*, which encodes for a protein essential in retinoic acid (RA) metabolism [105]. It has been previously shown that RA is capable of regulating the differentiation of epithelial cells, and more importantly, inhibit the growth of HeLa cells in vitro [106]. Although not validated in this study, it would be an exciting avenue to pursue. Other studies have focused on cellular miRNAs whose expression is regulated by E6 and E7, with predicted roles in cell proliferation, apoptosis, and differentiation [107,108].

## 3. Cellular Consequences Affected by E6 and E7

**Figure 3:**
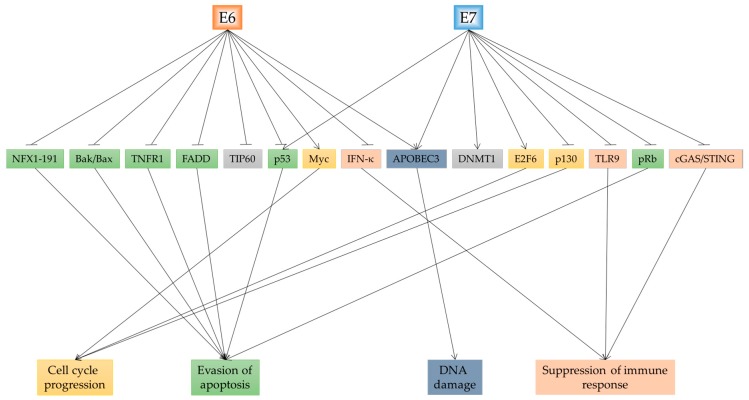
HPV oncogenes E6 and E7 target a plethora of cellular factors. This eventuates in cell cycle progression, evasion of apoptosis, DNA damage, and the suppression of the host cell immune response.

The infection of a cell with HPV can cause many cellular changes through mechanisms detailed above. Here, we will highlight the main cellular consequences which are driven by E6 and E7 and depicted in Figure 3.

### 3.1. Cell Cycle Changes and Growth Promotion

Evading checkpoints in the cell cycle is paramount for the process of tumorigenesis to occur. Thus, HPV targets key cellular factors which are involved in checkpoints in this crucial process. The most well-characterized targets of high-risk HPV—p53 and pRb are both key players in cell cycle dysregulation. Through the degradation of these two tumor suppressors, HPV-infected cells are able to bypass cell cycle checkpoints to maintain a proliferative, transformed phenotype. Researchers have utilized different molecular techniques to downregulate the expression of E6 and E7, such as CRISPR [109], transcription activator-like effector nuclease (TALENs) [110], artificial miRNAs [111], and siRNA [112], and have come to a similar conclusion that growth is markedly inhibited, and apoptosis/senescence is induced [113]. The HPV oncoproteins bring about these growth stimulatory changes through targeting and downregulating several key pro-apoptotic factors.

As previously mentioned, p53 is targeted by only high-risk types in a proteasome-dependent manner. Similarly, high-risk E7 binds to cullin 2 (CUL2) ubiquitin ligase complex to ubiquitylate and subsequently degrade pRb, initiating a cascade of downstream effects. The binding of E7 to Rb results in the de-repression the E2F family of transcription factors, and signals the cell to enter the S phase, and thus follow through with replication, despite insufficient resources in the cell.

Through the dysregulation of the proteins involved in these checkpoints, E6 and E7 can lead to uncontrolled cell growth and proliferation. The aberrant regulation of factors involved in cell cycle regulation in HPV-infected cells is so consistent that several groups have suggested using this as a diagnostic marker. Its potential was shown in a study of tissue microarrays by Conesa-Zamora et al. [114]. Using 144 fixed cervical tissue specimens, pathologists successfully detected p16, Ki-67, ProEx C (novel marker of MCM2 and TOP2A proteins), and p53 in high grade squamous intraepithelial lesions, of which p16, ProEx C, and Cyclin D1 correlated well with the severity of the lesion.

Through microarray analysis comparing normal human keratinocytes and differentiating cells harboring E6/E7, genes involved in G_2_–M phase transition were found to be upregulated, such as Plk1, Aurora-A, Nek2, and Cdk1, amongst others [115]. Due to Plk1′s important function in not only the G_2_–M transition, but also activation of cyclin B/Cdk1 and centrosome maturation, and regulation of the anaphase-promoting complex [116,117,118], the authors explored the mechanism of regulation of Plk1. Through mutational analyses, it was shown that Plk1′s regulation was dependent on E6′s ability to degrade p53, as well as E7′s repressive effect on pRb.

*Myc* is inarguably the most potent oncogene in the human cell, regulating the expression of 10–15% of cellular genes [119], and is involved in cell proliferation, apoptosis, and cellular transformation. *Myc* has been shown to be activated in more than half of cancer cases, and therefore, its overexpression is now accepted as one of the hallmarks of cancer (reviewed in [120]). *Myc* overexpression in cervical cancer is no exception, and the overexpression is brought about mainly by amplified gene expression, and detectable by PCR of cervical tissue scrapes of normal cervical tissue and HPV-infected tissues [121]. It was also observed that *c-myc* copy number increased along with histological grade, indicating that *c-myc* has a role in cellular transformation [121]. It was shown through in situ hybridization experiments that in some cases, the integration site of the HPV genome was in the same chromosomal band as *c-myc* or *n-myc*, 8q24, and 2p24, respectively, and the integration resulted in the cellular oncogene being structurally altered and/or amplified [122]. However, other studies have shown that the HPV integration site is not the only factor involved in *Myc* overexpression and thus, the mechanism of *Myc* amplification still remains to be elucidated.

In addition to HPV being a factor which led to the amplification of the locus, E6/E7 have also been found to regulate both the expression and the activity of Myc through several distinct mechanisms. Although both high and low-risk HPV can bind to Myc, only high-risk HPV can activate Myc, providing evidence for differences between high and low-risk phenotype [123]. Firstly, it has been shown that E6 is involved in the phosphorylation of Myc, increasing its stability in the cell [124]. However, this only occurs to a subpopulation of Myc, with different subpopulations of Myc having different stability and functions [125]. The exact mechanism of the phosphorylation, however, still stands to be confirmed.

Secondly, it was shown via co-immunoprecipitation assays that E6 and Myc are part of a complex, and is able to have functional consequences, such as the activation of *TERT* [126]. Thirdly, it was shown that E7 can bind to and activate c-Myc-mediated transcription activity [123]. Although both high and low-risk HPV E7 was shown to bind to c-Myc, only high-risk E7 can enhance c-Myc transcription activation. Using the *TERT* promoter as a model, the authors utilized immunoprecipitation assays to demonstrate that c-Myc bound to the *TERT* promoter in a manner dependent on E7 [123]. Treatment with Myc antagonists significantly abrogated E6-mediated transcription of *TERT* [124]. Fourthly, research has shown alternative methods in which E6 can utilize Myc to upregulate *TERT* expression. Zhang et al. showed that in addition to binding to Myc, E6 can also bind directly to Max, although only in the presence of Myc, suggesting that E6 can bind to Max when it is part of the Myc/Max heterodimer [124]. The final method in which HPV can regulate Myc is in a manner reverse to the abovementioned mechanisms. It has been shown that the family of Myc proteins also have a growth inhibitory role by inducing apoptosis when cell proliferation is inhibited [127]. It is hypothesized that through its degradation of Myc, E6 is able to evade Myc-driven apoptosis to maintain viral infection and proliferation [128]. However, this does not occur very often since tumor cells are mostly in their proliferative stage.

*TERT* is one of the central regulators in cancer, its expression is key for continued proliferation and to evade replicative senescence. As such, it is targeted and activated by HPV oncoproteins by several different methods. The promoter of *TERT* contains binding sites for the transcription factors Myc and Sp1, and it was shown that when either Myc or Sp1 binding sites were mutated, there was marginal decrease in *TERT* expression [129]. However, when all Myc and Sp1 binding sites were mutated, there was complete ablation of *TERT* expression. As described above, E6 and Myc have a very intricate relationship in the cell, and cooperatively, E6 and Myc are able to activate the *TERT* promoter [130]. It was also recently demonstrated that TIP60, destabilized by E6, acetylates Sp1 and ablates its binding to the *TERT* promoter [131]. In the presence of E6, TIP60 is targeted for proteasomal degradation by EDD1 [94], allowing Sp1 to bind to the *TERT* promoter, and therefore, leading to its activation. Secondly, NFX1-91, a repressor of *TERT*, has been shown to be a substrate of E6/E6AP [132,133]. Through the degradation of NFX1-91 in a proteasome-dependent manner, *TERT* expression is de-repressed in the presence of E6 and E6AP. Thirdly, it was demonstrated that E6 was able to post-translationally modify the histones at the promoter of *TERT*, the overexpression of which led to an increase in the activating H3K4Me3 mark with a concomitant decrease in the repressive H3K9Me2 mark [124]. Further ChIP assays also demonstrated that in cells overexpressing E6, there was increased serine 2 phosphorylation of Pol II at the *TERT* promoter, indicating increased transcription of *TERT.* A separate study by McMurray et al. demonstrated that in the absence of E6, a repressive complex containing USF1 and/or USF2 was localized on the *TERT* promoter, which was demonstrated by ChIP assays [134]. However, when E6 was transiently introduced into the system, this repressive complex was displaced from the promoter region, and Myc subsequently bound to the E-boxes on the *TERT* promoter. This led to a concomitant increase in TERT activity. However, the exact mechanism in which E6 affects the binding of these regulatory factors are not known.

### 3.2. Evading Apoptosis

Apoptosis is the scheduled death of the cell, and often occurs when the cell accumulates DNA damage. There are several methods in which E6 and E7 can block apoptosis. Although the HPV oncogene E7 sensitizes cells to p53-mediated apoptosis through the abrogation of pRb, this is overcome by E6-mediated degradation of p53.

Bak and Bax are members of the Bcl-2 protein family which have the canonical function of inducing apoptosis via the mitochondrial pathway. Upon sensing a variety of stress signals, Bak and Bax change conformation and assemble into oligomeric complexes in the mitochondrial outer membrane to form pores in the outer membrane, which subsequently lead to cellular apoptosis (reviewed in [135]). As key pro-apoptotic factors in the cell, it is only natural that Bak and Bax are targeted by both high and low-risk HPV E6, albeit via different mechanisms. Bak is targeted by both high and low-risk HPV types independent of p53 [136]. Interestingly, Bak was found to interact with both E6 and E6AP independent of each other, suggesting that Bak is a cellular target of E6AP, which is augmented by the presence of E6 [136]. The interaction of E6 with Bak is functional, where HPV18 E6 was shown to inhibit Bak-induced apoptosis in an HPV-negative cell line in the presence of exogenous E6. Although Bax has also been shown to be a target of E6, this interaction is mediated by p53 [137]. Upon the silencing of high-risk E6 via RNA interference (RNAi), p53 levels are rescued, reactivating the *PUMA* promoter, thus allowing Bax to be activated and translocated to the mitochondrial membrane.

Interestingly, studies have shown that E7 alone can induce apoptosis in a manner dependent on tumor necrosis factor [138]. This is hypothesized to be due to E7′s ability to stabilize the p53 protein which is abrogated in the presence of HPV E6 [139,140]. It was shown that E7 stabilizes p53 through a mechanism independent of p19 (ARF) [141]. The repressive complex DREAM (dimerization partner, RB-like, E2F4, and MuvB) is known to be downstream of p53, and has also been identified as a substrate of E7 [142,143,144]. The main function of the DREAM complex is to repress genes during quiescence. Upon re-entry into the cell cycle, DREAM complex, together with p130 and the repressive E2F4, dissociates from the promoter regions of cell cycle genes, and allows activating machinery to bind. DREAM is regulated by p53, and by binding to a myriad of different regions, such as cycle-dependent elements (CDEs), cell cycle genes homology regions (CHRs), CHR-like elements (CLE) and E2F sites, the DREAM complex is directed to promoters of cell cycle genes, leading to its repression [145,146]. It was shown that HPV16 E7 can bind to p130 of the DREAM complex via its LxCxE motif, resulting in proteasomal degradation, and therefore, abrogation of the DREAM complex [147]. Transcriptional repression mediated by DREAM opens up another avenue of p53 transcriptional activation, as p53 is canonically known to have only an activating function. One example of a gene of whose expression was de-repressed in a manner dependent on E7 and DREAM is *PLK4*, repressed by the p53–p21–DREAM complex. However, in the presence of E7, its expression is de-repressed [148].

This mechanism of E7 regulating cell cycle genes was verified when Fischer et al. analyzed publicly available gene expression datasets and found that the DREAM genes were the main group of genes deregulated by E7 [149]. This disruption was found to be indispensable for cell cycle progression in cervical cancer cells [142]. Interestingly, E7 can also target cyclin-dependent kinase (CDK) inhibitor p21, a crucial target of p53 required for its activity [150,151].

Inflammatory cytokines, such as TNFα, are secreted upon sensing of a viral infection, and this activates death receptors on the surface of the cell, such as TNF receptor 1 (TNFR1), TNF-related apoptosis-inducing ligand (TRAIL) receptors, and FAS. Through binding to TNFR1, E6 can inhibit the formation of the death-inducing signaling complex, blocking apoptosis from occurring [152]. Further, E6 also binds to protein FAS-associated protein with death domain (FADD) and caspase 8 to block downstream cell death [153,154].

### 3.3. Immune Response

Since HPV are infectious agents, it makes perfect evolutionary sense that one of the abilities of HPV is to disrupt innate immunity, since it is the first line of host defense against pathogenic infections. Viral nucleic acids (such as HPV) are sensed by pathogen recognition receptors (PRRs), such as TLR9 [155]. Canonically, TLR9 is able to recognize unmethylated CpG motifs on double-stranded DNA from viruses such as HPV [156], EBV [157], and HSV [158]. This initiates a cascade that eventually leads to the activation of host immune defense via the production of type I IFN and pro-inflammatory cytokines [159]. Research has recently shown that HPV16 is able to repress TLR9 transcription through the HPV oncoprotein E7, forming an inhibitory transcriptional complex comprising of NF-κBp50–p65 and ERα [160]. This complex recruits the histone demethylase JARID1B and histone deacetylase HDAC1 to the TLR9 promoter, resulting in a repression of its transcription. This is biologically significant, as the downstream interferon induction was negatively affected, muting the cellular effect of the viral infection.

A recent development in this field was the discovery that HPV oncogene E7 binds to STING, an adapter protein downstream of the intracellular DNA sensor, cGAS [161]. The cGAS–STING pathway is crucial in the detection of intracellular DNA and the initiation of the immune response in response to viral infection. The authors found that via the LXCXE motif on the E7 protein, the same motif involved in the degradation of pRb, E7 is able to antagonize DNA sensing, and therefore suppress an immune response post-infection.

Genes further downstream in the immunity signaling cascade were also observed to be regulated by HPV, such as the network of genes correlated with IL-1β [162]. These chemotactic and pro-inflammatory genes were discovered when a genome-wide screen was performed on undifferentiated keratinocytes containing episomal copies of high-risk HPV16 and 18. Along the same lines, HPV16, 18 and 31 were also found to repress the expression of genes involved in IFN signaling (*STAT1*), antiviral genes (*IFIT1* and *MX1*), pathogen recognition receptors (*TLR3*, *RIG-I*, and *MDA5*), and pro-apoptotic genes (*TRAIL* and *XAF1*) [163]. This was mediated by IFN-κ, found to be downregulated by E6 with the promoter of IFN-κ found to be methylated by DNMT1, recruited by E6 [164].

### 3.4. DNA Damage

Genomic instability is a hallmark of cancer cells, and malignant cells driven by HPV is no exception. It has been shown that through multiple mechanisms, HPV oncogenes E6 and E7 are able to independently cause DNA damage and chromosomal aberrations. This was shown through DNA breakage detection-fluorescence in situ hybridization (DNA-FISH) which detects DNA damage on a global, unbiased level [165]. Upon silencing of E6 and E7 in HeLa cells with lentiviral shRNA, there was a significant decrease in DNA damage, confirmed by alkaline comet assay. One mechanism that brings about chromosomal instability is stress during DNA replication, also known as replication stress. In the presence of HPV infection, E7 targets pRb for degradation, and this forces the cell to proceed with replication, despite an insufficient pool of nucleotides in the cell [166]. This eventuates in replication stress, and thus, genome instability. This phenotype was rescued when nucleosides were exogenously provided to HPV-infected cells, or by activating *c-myc*, which signals the increased transcription of nucleotide biosynthesis genes.

Another mechanism of genomic instability in malignant tumors is centrosome abnormalities, which potentially leads to defective/multiple mitotic spindle pole formations, resulting in chromosomal mis-segregation and genomic instability [167]. HPV16 E7 has been shown to induce abnormal centrosome synthesis, while E6 from the same type brings about nuclear atypia (multiple irregular nuclei) and accumulation of centrosomes [168]. Interestingly, the mechanism that E6 and E7 utilizes are disparate, from the simple observation that the overexpression of E7 results in an almost immediate increase in centrosomes, while centrosome abnormalities driven by E6 were only observed after several weeks in culture [169]. The mechanisms were later elucidated, and demonstrated that it is through the degradation of pRb by E7 that results in aberrant centriole synthesis [168]. Additionally, expression of both HPV16 E6 and E7 overwhelmed the spindle checkpoint control, allowing cells to enter into anaphase despite having multiple spindle poles [170]. The authors also found that HPV16 E7 could induce anaphase bridge formation, which typically occur after extensive chromosomal breaks, as well as inducing PARP formation, independent of E6.

One other target of high-risk E6/E7 is apolipoprotein B mRNA editing enzyme catalytic-polypeptide-like proteins 3 (APOBEC3), a family of cytidine deaminase proteins. The APOBEC3 family consists of seven different proteins, of which two are of interest in the field of HPV-induced cancers. APOBEC3A and APOBEC3B have been found to be highly expressed in HPV-positive samples, regulated by both E6 and E7, which will be detailed later [171,172]. The primary function of APOBEC3A/B enzymes is in the deamination of cytosine residues—that is, removing a crucial amino group from cytosine, converting it to an uracil. This occurs in both cellular genome and the viral genome, which eventually results in mutations (reviewed in [173]). APOBEC3-dependent mutagenesis on the viral genomes often result in the inhibition of replication, such as in the case of HIV-1 [174,175,176] and HPV [171]. Despite this, expression of APOBEC3 was found to be upregulated in HPV-positive samples [171,172]. This occurs through several different methods. Firstly, through the degradation of p53, HPV16 E6 upregulates TEAD1/4, a family of transcription factors involved in the regulation of multiple cellular targets, of which APOBEC3B is one [177]. Through ChIP experiments, the authors demonstrated that in normal immortalized human keratinocytes (NIKs) exogenously expressing E6, there was higher localization of TEAD4 on the promoter of APOBEC3B, resulting in increased transcription of APOBEC3B [177]. The second mechanism in which HPV is shown to result in increased levels of APOBEC3 is through the binding of HPV16 E7 to APOBEC3 via the CUL2 binding motif on APOBEC3A [178]. The formation of this complex prevented the degradation of APOBEC3A, and interestingly, despite this binding, APOBEC3A enzyme was found to retain its catalytic activity.

The dual function of APOBEC3A and APOBEC3B in restricting HPV, yet causing somatic mutations is probably one of the captivating reasons why researchers have focused on studying APOBEC. Interestingly, it has been found that small DNA viruses, HPV included, have an underrepresentation of TC dinucleotides [179], which are the substrates of the target-specific APOBEC3A enzymes. As such, HPV is partially resistant to the viral restriction acted upon by APOBEC3A and APOBEC3B. By upregulating these two enzymes, E6 and E7 are, instead, able to cause cancer mutagenesis. As an example, mutations in the oncogenic driver *PIK3CA* gene were found to be more prevalent in HPV-positive head and neck cancers (HNCs) compared to HPV-negative patients [180,181]. Further analyses revealed that all of the *PIK3CA* mutations in HPV-positive HNCs were caused by GA-to-AA mutations, while only half were caused by the same mutations in HPV-negative HNCs.

## 4. Therapeutics against HPV

### 4.1. Vaccinations

In June 2006, the US Food and Drug Administration (FDA) approved the first ever cervical cancer vaccine, Gardasil [182]. This vaccine contains virus-like particles (VLP) of HPV types 6, 11, 16, and 18, and was intended for females 9–26 years of age to prevent cervical cancer, and also HPV types that cause non-malignant lesions. This has been proven to be highly effective, reducing the incidence of genital warts in Australia from 12% to 5% in women, and 12% to 9% in heterosexual men, the latter attributed to lower exposure to the virus, since their sexual partners were no longer carriers of the virus [183]. As such, in 2013, Australia was the first country to begin vaccinating their male population in an effort to curb the transmission of HPV by males [184]. A second vaccination against HPV was approved by the FDA, Cervarix, a bivalent vaccine containing VLPs of high-risk types 16 and 18 (reviewed in [185]). The third vaccine to be approved by the FDA is 9-valent/Gardasil-9, which contains VLP covered by Gardasil, as well as five other oncogenic types-HPV type 31, 33, 42, 52, and 58. Clinical trials concluded in 2015 showed that when compared to Gardasil, there was lower incidence of high grade cervical, vulvar, and vaginal disease related to HPV type 31, 33, 45, 52, and 58 in the group with Gardasil-9. However, there was no difference in antibody responses to HPV types 6, 11, 16, and 18, of which both groups are vaccinated against [186].

In addition, there is an ongoing Phase III Clinical Trial funded by Shanghai Zerun Biotechnology Co. Ltd. (Clinical Trials Identifier: NCT02733068). This vaccine contains VLPs from L1 proteins from HPV types 16 and 18, and the trial is due to be completed in November of 2020.

### 4.2. Treatment

Although there have been numerous studies showing positive growth inhibitory effects upon the downregulation of the HPV driving oncogenes, most of the groups utilized RNAi, which had a transient effect on the cells. This temporary modulation is sufficient for mechanistic studies to be carried out, but insufficient to completely ablate E6 and E7 in vivo, to trigger apoptosis. Thus, mechanisms which utilize permanent genome editing capabilities are more promising for cervical cancer patients in the clinic.

There is an ongoing Phase I clinical trial by Hu and colleagues, in which they are utilizing TALEN and CRISPR/Cas9 targeting HPV16 E6/E7 or HPV18 E6/E7 to treat cervical cancer patients [109,110]. This study is expected to conclude in January of 2019 (Clinical Trial identifier: NCT03057912).

Additionally, other groups have developed molecules which block the activity of either E6, E7, or E6AP. This was made possible by the use of peptides, organic compounds, RNA molecules, nucleotide analog, small molecules, compound, zinc-ejecting inhibitor, heparin-like molecules or naturally-derived biopolymers (reviewed in [187]). There has also been a recent interest in the development of natural compounds derived from plants which have shown the ability to reduce the viral infection in patients with HPV-positive cervical cancer. In particular, a polyherbal formulation, administration of “Praneem” to patients positive for high-risk HPV16 for a period of 30 days led to non-detection of the virus via PCR-based methods [188].

Despite the great deal of research carried out, many of these therapies are still years away from making it into the clinic, and most late stage cervical cancer patients resort to hysterectomy and chemoradiation as treatment options with the most promise. Recently, a new targeted therapy was developed, which aims to curb the process of angiogenesis. Bevacizumab (Avastin^®^) is a monoclonal antibody against VEGF, the treatment of which has shown great promise in several types of cancer [189,190]. Clinicians have begun to include Bevacizumab into their treatment regime in combination with existing approved drugs.

## 5. Conclusions

HPV has evolved over the years to target many aspects of the host machinery, affecting ubiquitin ligases, epigenetic writers, miRNA, splicing regulators, amongst others. Through these interactions, the potent HPV oncogenes E6 and E7 are able to exert their effects in the cell to affect multiple cellular pathways. They are able to do so aside from the well-known targets p53 and pRb. We have shown that HPV targets major cellular pathways in multiple ways, creating “back-up plans” for itself to ensure that the cellular pathways are, indeed, dysregulated. The resultant effect is the continued proliferation of the host cell and replication of the virus to infect neighboring cells, thus contributing to carcinogenesis. Although there have been limited developments in targeted therapies, there are several vaccines in the market which are able to prevent the development of high-risk HPV infection.

## Abbreviations

AS	Angelman syndrome
APOBEC3	apolipoprotein B mRNA editing enzyme catalytic-polypeptide-like proteins 3
BGS	bisulfite genome sequencing
BTB	BR-C, ttk and bab
CARM1	co-activator associated arginine methyltransferase 1
CDEs	cycle-dependent elements
CDK	cyclin-dependent kinase
CHRs	cell cycle genes homology regions
CLE	CHR-like elements
CUL2	cullin 2
DNA-FISH	DNA breakage detection-fluorescence in situ hybridization
DREAM	DP, RB-like, E2F4, and MuvB
DUBs	deubiquitinating enzymes
E6AP	E6-associated protein
EBV	Epstein–Barr virus
FADD	FAS-associated protein with death domain
FDA	Food and Drug Administration
GPCA	*Gaussia princeps* luciferase protein complementation assay
HAT	histone acetyltransferase
HBV	hepatitis B virus
HC2	hybrid capture 2
HCV	hepatitis C virus
HDAC	histone deacetylases
HECT	homologous to E6AP carboxyl-terminus
HFKs	human foreskin keratinocytes
HMTs	histone methyltransferases
HNCs	head and neck cancers
HNSCC	head and neck squamous cell carcinoma
HPV	human papillomavirus
HSV	herpes simplex virus
HTLV-1	human T-lymphotropic virus-1
KSHV	Kaposi’s sarcoma-associated herpesvirus
LCR	long control region
MCV	Merkel cell polyomavirus
miRNAs	microRNAs
MSDK	methylation-specific digital karyotyping
MVP	methylation variable positions
NIKs	normal immortalized human keratinocytes
NLS3	nuclear localization signal 3
P/CAF	p300/CBP-associated factor
PBM	PDZ-binding motif
PKA	protein kinase A
pRb	retinoblastoma protein
PRC	polycomb repressive complex
PRMT1	protein arginine methyltransferase 1
PRRs	pathogen recognition receptors
RNAi	RNA interference
SCC	squamous cell carcinoma
SET7	SET domain containing lysine methyltransferase 7
SR	splicing regulatory
SSA	single-strand annealing
TALEN	transcription activator-like effector nuclease
TNFR1	TNF receptor 1
TRAIL	TNF-related apoptosis-inducing ligand
TRAM	transcriptional adapter motif
UPS	ubiquitin–proteasome system
VLP	virus-like particles
